# Association of *SLC6A3* variants with treatment-resistant schizophrenia: a genetic association study of dopamine-related genes in schizophrenia

**DOI:** 10.3389/fpsyt.2023.1334335

**Published:** 2024-02-27

**Authors:** Masanobu Kogure, Nobuhisa Kanahara, Atsuhiro Miyazawa, Yuki Shiko, Ikuo Otsuka, Koichi Matsuyama, Masayuki Takase, Makoto Kimura, Hiroshi Kimura, Kiyomitsu Ota, Keita Idemoto, Masaki Tamura, Yasunori Oda, Taisuke Yoshida, Satoshi Okazaki, Fumiaki Yamasaki, Yusuke Nakata, Yoshinori Watanabe, Tomihisa Niitsu, Akitoyo Hishimoto, Masaomi Iyo

**Affiliations:** ^1^Department of Psychiatry, Chiba University Graduate School of Medicine, Chiba, Japan; ^2^Division of Medical Treatment and Rehabilitation, Center for Forensic Mental Health, Chiba University, Chiba, Japan; ^3^Doujin-kai Kisarazu Hospital, Kisarazu, Japan; ^4^Biostatistics Section, Clinical Research Center, Chiba University Hospital, Chiba, Japan; ^5^Department of Psychiatry, Kobe University Graduate School of Medicine, Kobe, Japan; ^6^Douwa-kai Chiba Hospital, Funabashi, Japan; ^7^Shoushin-kai Mobara Mental Hospital, Mobara, Japan; ^8^Chiba Psychiatric Medical Center, Chiba, Japan; ^9^Department of Psychiatry, Kameda Medical Center, Kamogawa, Japan; ^10^Gakuji-kai Kimura Hospital, Chiba, Japan; ^11^Department of Psychiatry, School of Medicine, International University of Health and Welfare, Narita, Japan; ^12^Choshi-kokoro Clinic, Choshi, Japan; ^13^Department of Cognitive Behavioral Psychology, Chiba University Graduate School of Medicine, Chiba, Japan; ^14^Himorogi Psychiatric Institute, Tokyo, Japan

**Keywords:** antipsychotic, dopamine, psychosis, single nucleotide polymorphism, variable number of tandem repeats

## Abstract

**Background:**

Most genetic analyses that have attempted to identify a locus or loci that can distinguish patients with treatment-resistant schizophrenia (TRS) from those who respond to treatment (non-TRS) have failed. However, evidence from multiple studies suggests that patients with schizophrenia who respond well to antipsychotic medication have a higher dopamine (DA) state in brain synaptic clefts whereas patients with TRS do not show enhanced DA synthesis/release pathways.

**Patients and methods:**

To examine the contribution (if any) of genetics to TRS, we conducted a genetic association analysis of DA-related genes in schizophrenia patients (TRS, *n* = 435; non-TRS, *n* = 539) and healthy controls (HC: *n* = 489).

**Results:**

The distributions of the genotypes of rs3756450 and the 40-bp variable number tandem repeat on *SLC6A3* differed between the TRS and non-TRS groups. Regarding rs3756450, the TRS group showed a significantly higher ratio of the A allele, whereas the non-TRS group predominantly had the G allele. The analysis of the combination of *COMT* and *SLC6A3* yielded a significantly higher ratio of the putative low-DA type (i.e., high COMT activity + high SLC6A3 activity) in the TRS group compared to the two other groups. Patients with the low-DA type accounted for the minority of the non-TRS group and exhibited milder psychopathology.

**Conclusion:**

The overall results suggest that (*i*) *SLC6A3* could be involved in responsiveness to antipsychotic medication and (*ii*) genetic variants modulating brain DA levels may be related to the classification of TRS and non-TRS.

## 1 Introduction

Approximately 30% of individuals with schizophrenia do not respond well to any standard antipsychotics, even at a sufficient dose or sufficient duration; these patients are defined as having treatment-resistant schizophrenia (TRS) (1, 2). Patients with TRS generally continue to represent severe psychiatric symptoms along with an unstable clinical course over the long term, leading to very poor outcomes. Several studies have suggested that TRS patients should be understood as those who do not respond well to antipsychotic medications, meeting the diagnostic criteria of TRS from the early treatment period (3–5). Numerous risk factors for TRS such as younger onset (6–9), poor premorbid social functioning (10–12), and autistic traits (13, 14) suggest that psychiatric abnormalities may have already begun prior to the onset of psychosis.

Magnetic resonance spectroscopy (MRS) studies have demonstrated some differences in GABAergic and glutamatergic networks between patients with TRS taking clozapine and those who are not taking clozapine (15–19). Clozapine, the only approved agent for TRS, is presumed to have effects on GABAergic and glutamatergic systems, which may be associated with its high efficacy (20, 21). These findings indicate that (*i*) patients with TRS may have a pathology that differs from that of non-TRS patients, and (*ii*) abnormalities in GABAergic and glutamatergic systems could be involved in the pathogenesis of TRS (5, 22, 23).

Alterations in the dopamine (DA) system have also been documented in patients with TRS. Several positron emission tomography (PET) studies reported that the DA synthesis capacity of TRS patients was not enhanced (i.e., it was comparable to that of healthy controls), in contrast to patients who responded to an antipsychotic with an enhanced synthesis and release of DA (24, 25). Other PET studies also suggested that patients with higher DA levels respond better to antipsychotic medication (26–28). An investigation by Amato et al. (29) using animal models indicated that an organically high DA state in the synaptic cleft is essential for the efficacy of antipsychotic drugs, and that the tyrosine hydroxylase (TH) enzyme and dopamine transporter (DAT) proteins could play important roles in the efficacy of antipsychotics.

Several genetic studies have focused on single nucleotide polymorphisms (SNPs) of candidate genes [e.g., dopamine D2 receptor gene (*DRD2*)] in patients with TRS (30–34), but nearly all of them failed to identify definite variant(s) relating to TRS pathology, partly due to the studies’ relatively small sample sizes. An increasing number of polygenic risk score (PRS) analyses of TRS with genome-wide association study (GWAS) data have been published, but their results have been inconsistent: some reports showed that TRS patients exhibited significantly higher PRS values compared to non-TRS patients (35–38) whereas others denied this finding (39, 40). These inconsistencies could potentially be explained by the diagnosis of TRS based on the use of clozapine as a surrogate marker and the varying rates of TRS patients in the discovery cohort used to create the PRS threshold (35, 36). Given the rich evidence of a relationship between the DA synthesis capacity and responses to antipsychotic medication, we speculated that compared to consortium genome samples, a study of TRS patients whose cases were collected at the same time as their clinical assessments (including accurate medication histories) would be more beneficial to detect variants on genes that are relevant to the response to antipsychotics.

In order to gain a deeper understanding of the mechanisms underlying TRS, we conducted the present study of patients with and without TRS to investigate molecules that modulate the amount of DA in the synaptic cleft (i.e., synthesis, degradation, and reuptake) from a genetics viewpoint. We also evaluated the status of gene variants that are known to be functionally relevant to the dopaminergic system and then compared the findings in the TRS patients with those in the non-TRS patients and a group of healthy controls.

## 2 Subjects and methods

### 2.1 Subjects

We analyzed the cases of a total of 974 patients who were treated at one of several psychiatric hospitals in mainly Chiba prefecture, which is east of Tokyo. They were diagnosed as having schizophrenia or schizoaffective disorder by at least two experienced psychiatrists based on the Diagnostic and Statistical Manual of Disorders IV-TR or the 5th edition (DSM-5). Patients who met the criteria of substance abuse were excluded from the study. A total of 489 healthy controls also participated in the study. They were confirmed to have no history of psychiatric disorders of their own or among family members. All participants were Japanese.

At the blood sample collection, the subjects were provided a detailed explanation of the study and gave their written informed consent to participate in the study. For a patient who was judged to be compromised in understanding the study due to psychiatric symptoms, the consent was obtained from his/her family member. The study was approved by the ethics committees of Chiba University Graduate School of Medicine and the other psychiatric hospitals that participated in the study. The study was conducted in accord with the Declaration of Helsinki.

### 2.2 Diagnosis of treatment-resistant schizophrenia

The diagnosis of TRS was made based on both the evaluation by the patient’s attending physician and the patient’s chart record. The diagnostic criteria for TRS followed the criteria of the Clozapine Patient Monitoring Service (CPMS), which defines patients with TRS as those who have not responded adequately [i.e., never reaching a Global Assessment of Functioning (GAF) score > 41 points in the 12 months prior to the blood sampling] to at least two different antipsychotic medications with a ≥ 600 mg chlorpromazine-equivalent (CP-eq.) dose and ≥ 4 weeks’ treatment with each medication. Patients who met the “intolerance to antipsychotic medication” criterion in the CPMS criteria were excluded from the study. The patients’ levels of adherence to their medications was also assessed as part of the determination of whether they met the criteria for TRS. Those who clearly did not meet the TRS criteria were judged as belonging to the non-TRS group in this study. The patients for whom sufficient information was unavailable were removed from the analysis.

### 2.3 Selected variants

As shown in Table 1, eight functional variants of genes relevant to DA synthesis, metabolism, and reuptake and DRD2 were selected for analysis, following previous genetic studies.

**Table 1:** Eight polymorphisms (SNP and VNTR) examined in the present study.

SNP	Variant and position	Evidence
*TH*		
rs10770141	G824A: 5′-UTR	Individuals who have the minor allele A have been reported to have 30–40% higher gene expression of TH compared to subjects without an A allele (41).
rs6356	Val(A)81Met(G): Exon3	This SNP is located within the regulatory domain of the *TH* gene (42). There is a recent report that the A allele of this SNP is associated with a high expression of the *TH* gene in the hippocampus and nucleus accumbens (43).
*DRD2*		
rs1800497	Taq1A with the reference allele [A] named “A1” and the substituted allele G named “A2”: Exon1 on *ANKK1* gene:	PET studies indicated that the A1 allele is associated with a 40% reduction in DRD2 expression in the striatum (44–46). Some research groups have reported that this variant was significantly related to treatment responsiveness and cognitive function in schizophrenic patients in meta-analyses (47, 48).
rs6275	A939G: Exon7	There are several reports that schizophrenia patients with the AA genotype have particularly poor attention and executive function (49–51).
*COMT*		
rs4680	Val158Met: Exon3	This polymorphism affects the activity of the COMT enzyme, with Met (A allele) homozygotes having 40% less enzyme activity than Val (G allele) homozygotes, leading to increased DA levels in the prefrontal cortex (PFC) and anterior cingulate cortex (ACC) (52, 53).
*SLC6A3*		
rs3756450	A > G: 5′-UTR	Recently, the genetic makeup of the 5′-UTR was found to affect the expression of DAT (54). The alteration allele G decreased the expression compared to the reference allele A (55).
rs420422	G > A: Intron3	The region including this SNP is reported to be related to splicing variants of DAT (56).
40-bp VNTR	3′-UTR	This VNTR was extensively studied since this can affect the expression of DAT, which was not clarified yet (57–59). The most common forms of VNTR are the 9- and 10-repeat types.

### 2.4 Genotyping of SNPs and the analysis of VNTR polymorphisms

Genomes were extracted from each subject’s blood sample with the use of the QIAamp DNA Blood Mini Kit (250) (Qiagen, Valencia, CA, United States). The genotype of each SNP was determined by a TaqMan probe assay (Applied Biosystems, Foster City, CA, United States) using an ABI PRISM 7300 real-time polymerase chain reaction (PCR) system. This was done at 95°C for one 10-min cycle, followed by 40 cycles of 95°C for 15 s and 60°C for 60 s.

For the amplification of the 40-bp variable number tandem repeat (VNTR) on Solute Carrier Family 6 Member 3 (dopamine transporter) gene (*SLC6A3*), the following primer sequences were used: upstream, 5′-TGTGGTGTAGGGAACGGCCTGAGA-3′; downstream, 5′-TGTTGGTCTGCAGGCTGCCTGCAT-3′ (60). The PCR procedure was followed by a one-cycle denaturation step at 95°C for 1 min, followed by 35 cycles of denaturation at 95°C for 15 s, annealing at 55°C for 15 s, extension at 72°C for 30 s, and finally an extension step at 72°C for 7 min. Then, 2 μL of PCR products were run on a 2% agarose gel, and the repeat allele sizes were quantified with a transilluminator: 7-repeat (330 bp), 8-repeat (370 bp), 9-repeat (410 bp), 10-repeat (450 bp), and 11-repeat (490 bp).

### 2.5 Statistical analyses

The demographic characteristics of the healthy controls (HC), schizophrenia (SCH) group, and patients with and without TRS were compared using Student’s *t*-test or an ANOVA for continuous variables, and the χ^2^ test for categorical variables. We used the same statistical methods to compare the allelic and genotype distributions of the eight studied SNPs between the schizophrenia subgroups and the HC group. As *post hoc* tests, Bonferroni correction was used for the ANOVA and a residual analysis was used for the χ^2^ test. To explore the potential impact of each SNP on the classification of TRS or non-TRS, we conducted a multivariate logistic analysis in which we used the group (TRS, non-TRS, or HC) as the response variable and the genotype of each SNP as the explanatory variables with patient’s age and sex as covariates.

The statistical significance level was set at *p* < 0.05, with the exception of the comparisons of SNP distribution. For the analysis of SNP distributions, a Bonferroni correction for multiple comparisons was applied, and the statistical significance level was thus set at *p* = 0.00625 (=0.05/8). All statistical analyses were performed using SPSS ver. 22.0 software (IBM, NY).

## 3 Results

The SCH group had a significantly higher proportion of females (49.8%) compared to the HC group (44.1%) (Table 2). The patients’ ages at the time of blood sampling were significantly higher in the SCH group compared to the HC group. There was also a significant difference in age among the three groups: the TRS group was the oldest, followed by the non-TRS group, with the youngest group being the HC group.

**Table 2:** Demographic characteristics of the healthy controls, schizophrenia group, and the patients with and without treatment-resistant schizophrenia.

Variable	HC group	SCH group	TRS group	Non-TRS group	Statistical values	
	*n* = 489	*n* = 974	*n* = 435	*n* = 539	HC vs. SCH	HC vs. TRS vs. non-TRS
Sex: male/female, *n*	271/214^a^	487/484^a^	222/211	265/273	χ^2^ = 4.243, ***p* = 0.039**	χ^2^ = 4.633, *p* = 0.099
Age at blood sampling, years^*^	36.68 (15.20)	46.64 (14.87)	49.29 (14.43)	44.45 (14.88)	*t* = 11.946, ***p* < 0.001**	*F* = 85.105, ***p* < 0.001**
						TRS > non-TRS > HC

^*^These data are mean (SD).^a^The age at blood sampling was unknown for 19 patients in the schizophrenia (SCH) group and two in the healthy control (HC) group. TRS: treatment-resistant schizophrenia.

### 3.1 Univariate analysis

The univariate analysis detected no polymorphisms that showed deviant results from the Hardy–Weinberg equilibrium in the HC or SCH groups.

#### 3.1.1 SCH vs. HC

None of the seven polymorphisms other than the 40-bp VNTR showed significant differences in genotype or allelic distribution between the HC and SCH groups (Supplementary Table S1). Regarding the 40-bp VNTR, there was a significantly higher ratio of subjects with the 10X genotype in the SCH group compared to the HC group.

When additional analyses separating male and female subjects were conducted, there were no significant differences between the HC and SCH groups for any of the eight variants (data not shown).

#### 3.1.2 TRS vs. non-TRS vs. HC

There was a significant difference in rs3756450 in both the genotype and allelic distributions; the residual analysis showed a higher ratio of A alleles in the TRS group and a higher ratio of G alleles in the non-TRS group (Table 3). These results are the same as those obtained in the analyses that were conducted separately for the male and female subjects (the residual analysis showed a higher ratio of G allele in the non-TRS group, with *p* values of 0.00338 in the males-only analysis and 0.0418 in the females-only analysis), although there was no significant difference in the genotype-based analyses.

**Table 3:** Comparisons of the allelic and genotype distributions of the eight studied SNPs between the schizophrenia subgroups and the healthy control group.

SNP	Major/minor allele (MAF)^#^	Group	Genotype *n* (%)				Allele *n* (%)				OR	95% CI
			MM	Mm	mm	*p* value	M	m		*p* value		
*TH*												
rs10770141	G > A [A:0.068]	HC	359 [85.7]	57 [13.6]	3 [0.7]	0.696	775 [92.5]	63 [7.5]		0.837		
		Non-TRS	457 [86.2]	72 [13.6]	1 [0.2]		986 [93.0]	74 [7.0]			0.923	0.651–1.309
		TRS	371 [87.1]	52 [12.2]	3 [0.7]		794 [93.2]	58 [6.8]			0.910	0.621–1.301
rs6356	A > G [G:0.325]	HC	201 [49.4]	170 [41.8]	36 [8.8]	0.317	572 [70.3]	242 [29.7]		0.396		
		Non-TRS	238 [45.8]	235 [45.2]	47 [9.0]		711 [68.4]	329 [31.6]			1.094	0.896–1.335
		TRS	215 [52.1]	158 [38.3]	40 [9.7]		588 [71.2]	238 [28.8]			0.957	0.773–1.184
*DRD2*												
rs1800497	G > A [A:0.373]	HC	205 [45.7]	184 [41.0]	60 [13.4]	0.311	594 [66.1]	304 [33.9]		0.256		
		Non-TRS	214 [40.2]	239 [44.8]	80 [15.0]		667 [62.6]	399 [37.4]			1.169	0.971–1.407
		TRS	176 [41.1]	196 [46.5]	53 [12.4]		551 [64.4]	305 [35.6]			1.082	0.889–1.317
rs6275	A > G [G:0.432]	HC	176 [37.1]	215 [45.3]	84 [17.7]	0.287	567 [59.7]	383 [40.3]		0.183		
		Non-TRS	167 [31.5]	261 [49.2]	103 [19.4]		595 [56.0]	467 [44.0]			1.162	0.973–1.388
		TRS	148 [34.7]	211 [49.4]	68 [15.9]		507 [59.4]	347 [40.6]			1.013	0.893–1.223
*COMT*												
rs4680	G > A [A:0.317]	HC	205 [42.8]	210 [43.8]	64 [13.4]	0.288	620 [64.7]	338 [35.3]		0.310		
		Non-TRS	243 [44.9]	231 [43.2]	61 [11.4]		717 [67.0]	353 [33.0]			0.903	0.751–1.085
		TRS	192 [44.9]	198 [46.3]	38 [8.9]		582 [68.0]	274 [32.0]			0.864	0.710–1.050
*SLC6A3*												
rs3756450	A > G [G:0.469]	HC	133 [30.8]	210 [48.6]	89 [20.6]	**0.006**	476 [55.1]	388 [44.9]		**<0.001**		
		Non-TRS	112 [22.6]	250 [50.4]	134 [27.0]		474 [47.8]	518 [52.2]			1.341	1.117–1.620
		TRS	124 [30.8]	200 [49.8]	78 [19.4]		448 [55.7]	356 [44.3]			0.975	0.804–1.183
rs420422	G > A [A:0.392]	HC	152 [35.8]	202 [47.6]	70 [16.5]	0.330	506 [59.7]	342 [40.3]		0.119		
		Non-TRS	214 [41.6]	233 [45.3]	67 [13.0]		661 [64.3]	367 [35.7]			0.822	0.681–0.991
		TRS	157 [37.8]	200 [48.2]	58 [14.0]		514 [61.9]	316 [38.1]			0.910	0.748–1.107
			**10 10**	**10 X**	**X Y**		**10 repeat**	**9 repeat**	**Others**			
40-bp VNTR	10 > others [10: 0.93]^#^	HC	93 [91.2]	9 [8.8]	0 [0]	**0.004**	195 [95.6]	5 [2.5]	4 [2.0]	**0.023**		
		Non-TRS	79 [80.6]	19 [19.4]	0 [0]		177 [89.8]	8 [4.1]	12 [6.1]		**2.448**	1.086–5.518
		TRS	77 [77.8]	17 [17.2]	5 [5.1]		171 [86.8]	15 [7.6]	11 [5.6]		**3.294**	1.502–7.225

SNP, Single nucleotide polymorphism; VNTR, Variable number of tandem repeats.^#^From Sano et al. (60).

The results for the 40-bp VNTR also showed a significant difference, with a higher ratio of XY in the TRS group and a higher ratio of homozygotes with 10 10 in the HC group (Table 3). When 10-repeat, 9-repeat, or other repeat allele sizes were examined separately, we observed that the distribution differed significantly among the three groups (Supplementary Table S2). This was also true for the analysis of the females-only group, while there was no significant difference in the analysis for the male subjects. There were no significant differences in the genotype or allelic distribution for other polymorphisms in the entire series of subjects or in the separate analyses of males and females.

### 3.2 Multivariate logistic analysis

By applying the seven SNPs or the seven SNPs plus the 40-bp VNTR to multivariate logistic models, we searched for significant variants affecting the classification of the TRS and non-TRS patients.

The first model including the seven SNPs revealed that the GG genotype of rs1800497 was significantly less related to TRS, with an odds ratio (OR) of 0.640 (*p* = 0.045) using the HC group as the reference (Table 4). When the TRS group was compared with the non-TRS (reference) group, the GG genotype of rs3756450 showed protection against TRS (OR = 0.685, *p* = 0.044), whereas the AA genotype was related to a risk of TRS (OR 1.430, *p* = 0.043).

**Table 4:** Multivariate logistic regression analysis discriminating the TRS and non-TRS groups.

	TRS			non-TRS		
	OR	95%CI	*p* value	OR	95%CI	*p* value
Model including SNPs ①–⑦						
*Reference: HC*						
rs1800497 GG vs. GA	0.640	0.413–0.991	**0.045**	0.804	0.542–1.193	0.279
*Reference: non-TRS*						
rs3756450 GG vs. GA	0.685	0.475–0.989	**0.044**			
rs3756450 AA vs. GA	1.430	1.011–2.022	**0.043**			
Model including SNPs ①–⑦ and 40 bp-VNTR						
*Reference: HC*						
rs1800497 GG vs. GA	0.191	0.072–0.502	**0.001**	0.185	0.072–0.474	**<0.001**
rs4680 GG vs. GA	0.257	0.121–0.546	**<0.001**	0.285	0.137–0.592	**0.001**
*Reference: non-TRS*						
rs3756450 AA vs. GA	2.362	1.051–5.306	**0.037**			

The second model including the seven SNPs plus the 40-bp VNTR indicated that the GG genotype of rs1800497 and the GG(ValVal)genotype of rs4680 carried lower risks for TRS and non-TRS relative to the HC (reference) group, suggesting that there can be protective genotypes against schizophrenia *per se* (Table 4). When the non-TRS group was the reference group, the AA genotype of rs3756450 was significantly related to TRS, with an OR of 2.362 (*p* = 0.037).

### 3.3 The combination analysis with COMT and SLC6A3

We next examined the effect of the combination of rs4680 on Catechol-*O*-methyltransferase gene (*COMT*) and rs3756450 (*SLC6A3*), which we speculated may alter the activity of the two proteins and thus modulate the amount of DA in the synaptic cleft; our hypothesis was that patients with the combination of variants exhibiting a low-DA state would show a poorer response to antipsychotic medications. In this analysis, we defined patients with the combination of the GG (ValVal) of rs4680 and the AA of rs3756450 as those with a “low-DA combination” (in place of the actual measurement of DA levels) and the patients with other combinations as “others.”

The results revealed that the distribution of this genotype combination differed between the TRS and non-TRS groups: the TRS group more frequently had a low-DA combination and less frequently had other combinations (χ^2^ = 5.304, *p* = 0.021). These results were the same for the females-only analysis, whereas the male-only analysis showed no significant difference. Additional analyses of the TRS vs. non-TRS vs. HC groups supported these results, revealing that the low-DA combination was significantly less common in the non-TRS group (χ^2^ = 6.000, *p* = 0.0498) compared to the TRS group, whereas the distribution in the TRS and HC groups did not differ significantly (Table 5).

**Table 5:** Genotypic analysis of the *COMT* gene rs4680 combined with the *SLC6A3* gene rs3756450.

rs4680/rs3756450	HC group (*n* = 427)	non-TRS group (*n* = 495)	TRS group (*n* = 400)	Statistical values	
				*Post-hoc*	
GG / AA	54 (12.6)	43 (8.7)	54 (13.5)	χ^2^ = 6.000, ***p* = 0.0498**	GG/AA: TRS = HC > non-TRS
Others	373 (87.4)	452 (91.3)	346 (86.5)		Others: TRS = HC < non TRS

The data are number of subjects (%).

### 3.4 SLC6A3 and clinical parameters

We also examined the potential effects of the combination of rs4680 and rs3756450, rs4680 alone, rs3756450 alone, and the 40-bp VNTR on clinical indicators. We observed that in the TRS group, there were no significant effects of the combination of the two SNPs, rs4680, rs3756450, or 40-bp VNTR on any clinical measurements.

In the non-TRS group, the analyses of the two-SNP combination and rs3756450 showed significant differences in the subjects’ Clinical Global Impression-Severity (CGI-S) scale score, employment, and antipsychotic dosage: the patients with the low-DA combination among the combined two-SNP or G allele carriers of rs3756450 exhibited more severe psychopathology in terms of the CGI-S and employment and were being treated with higher doses of antipsychotics compared to the patients with other combinations and compared to the patients without the G allele (Table 6; Supplementary Table S3).

**Table 6:** Comparison of clinically related measures for rs3756450 G(+) carriers and G(−) carriers in the non-TRS groups.

Variables	rs3756450 G(+)	rs3756450 G(−)	Statistic values
	*n* = 387	*n* = 113	
Sex: male/female [*n*]	180/206	63/50	χ^2^ = 2.910, *p* = 0.088
Age at time of blood sampling [years]	45.26 (15.35)	44.09 (14.26)	*t* = 0.715, *p* = 0.475
Age at onset [years]	27.19 (10.35)	27.25 (9.09)	*t* = 0.046, *p* = 0.963
Duration of disease [years]	11.62 (9.79)	14.31 (11.31)	*t* = 1.902, *p* = 0.058
CGI-S	3.15 (1.07)	2.79 (0.97)	*t* = 2.610, ***p* = 0.010**
Number of hospitalizations [times]	1.45 (1.84)	1.31 (2.27)	*t* = 0.544, *p* = 0.587
Work experience following disease onset: Yes/No	120/104	52 / 21	χ^2^ = 7.046, ***p* = 0.008**
Employment at time of blood sampling: Yes/No	69/157	36 / 36	χ^2^ = 9.070, ***p* = 0.003**
ECT from onset of illness to time of blood sampling: Yes/No	6/231	3 / 74	χ^2^ = 0.389, *p* = 0.533
Clozapine medication from onset to present: Yes/No	0/235	0 / 76	-
Antipsychotic dose (CP-eq.) [mg]	477.9 (336.7)	331.2 (186.6)	*t* = 3.665, ***p* < 0.001**
Monotherapy with antipsychotic: Yes/No	185 / 51	68 / 10	χ^2^ = 2.894, *p* = 0.089

Cells with a value and parenthesis indicate mean (SD). Sex was unknown for one patient in the rs3756450 G(+) group.

Regarding both rs4680 and the 40-bp VNTR, there were no significant differences between polymorphisms or clinical measurements among the SCH, TRS, and non-TRS groups.

## 4 Discussion

Our findings demonstrated that the distributions of the genotypes of rs3756450 and the 40-bp VNTR of *SLC6A3* differed between the TRS and non-TRS groups in the following three ways: (*i*) regarding rs3756450, the A allele was significantly less abundant in the non-TRS patients and significantly more abundant in the TRS patients (Tables 3, 4). Concerning the 40-bp VNTR, the TRS group had significantly fewer carriers of 10-repeat homozygotes compared to the non-TRS and HC groups (Table 3; Supplementary Table S2). (*ii*) The analysis of the combination of *COMT* and *SLC6A3* revealed the TRS group’s significantly higher ratio of patients with the low-DA type (i.e., high COMT activity + high DAT activity) compared to both the HC and non-TRS groups (Table 5). (*iii*) In the non-TRS group, patients with the low-DA type were a minority and exhibited milder psychopathology compared to those with other genotype combinations (Supplementary Table S3; Table 6).

Several neuroimaging studies have indicated that the capacity to synthesize DA is not enhanced in patients with TRS and that a lower level of DA in the synaptic cleft may contribute to their nonresponsiveness to antipsychotics (24, 25). Our present results are the first to provide evidence that *SLC6A3* may be involved in the responsiveness to antipsychotic medication or the classification of TRS/non-TRS and that the specific genotype leading to the low-DA state could be related to patients with TRS.

*In vitro* studies have revealed that several variants spanning from the 5′-UTR to exon 1 on *SLC6A*3 affect the expression of DAT in a combined manner (61–63). These variants are in a relatively high-linkage disequilibrium (LD) relationship (64), and their alteration allele uniformly leads to lower expression relative to their respective reference allele (61, 64, 65). The same difference in expression was true for rs3756450: the alteration allele (A) showed lower expression (55, 65), except in a single study (62). The binding site with transcription factors is defined uniquely to the specific allele in the 5′-UTR on *SLC6A3*, which might be a reason for the differential expression by alleles (65).

Regarding the association with schizophrenia, two studies have demonstrated that rs3756450 was not the top-hit polymorphism of *SLC6A3*; however, it showed a highly significant signal in an association analysis between patients with schizophrenia and healthy subjects (55, 64). This finding was supported by a meta-analysis of the association between rs3756450 and schizophrenia (66). In the present study, this SNP was not significantly related to the patients with schizophrenia as a whole (Supplementary Table S1). However, when we divided the patients into TRS and non-TRS groups, a significant difference was observed: the non-TRS group had a significantly higher ratio of the G allele of rs3756450 (Table 3). In contrast, the TRS group did not differ from the HC group in the genotype or allelic distributions of rs3756450, but the TRS group had a significantly higher ratio of AA genotype carriers than the non-TRS group. In fact, the AA genotype was identified as the only risk factor in the multivariate logistic analysis including all other polymorphisms as independent variables (Table 4). These results suggest that in addition to the potential involvement of this SNP is might not only be involved in the vulnerability to schizophrenia (i.e., significant relationship with the non-TRS group), it may also be involved in the development of TRS following the onset of the disease.

Our present results showed a significantly higher ratio of the 9-repeat of the 40-bp VNTR in the TRS group compared to the non-TRS and HC groups (Supplementary Table S2). The 40-bp VNTR polymorphism in *SLC6A3* can affect its transcription (57, 67). Extensive *in vivo* and *in vitro* studies have obtained highly controversial findings regarding the effect of the 40-bp VNTR polymorphism in *SLC6A3* on DAT expression: higher expression in 10-repeat relative to 9-repeat (68–72); higher expression in 9-repeat compared to 10-repeat (73, 74); and no difference between them (75–78). This uncertain situation remains as of this writing, and there is some speculation that these variants are unlikely to be involved in schizophrenia (58, 59).

As our results concerning the 40-bp VNTR came from a small sample size, no conclusion can be drawn. However, the significant difference in the distribution of the 40-bp VNTR between schizophrenia subtypes provides an important insight into its relation to the responsiveness to antipsychotic medication among patients with schizophrenia, although two studies have denied the association of the 40-bp VNTR with responses to antipsychotics (79, 80). The regulation of the expression of DAT has not yet been clarified, and several regions such as the 5′-UTR (including rs3756450), the 3′-UTR (including the 40-bp VNTR), and other variants are thought to work together to determine expression levels (81). A complete understanding of the regulation of DAT expression is strongly desired.

In our additional analysis of the combined effects with the Val/Met polymorphism (rs4680) of *COMT* and rs3756450 of *SLC6A3*, which are suspected to more strongly affect the amounts of DA in the synaptic cleft, we observed that the TRS group’s frequency of this combination did not differ significantly from that of the HC group, but the ratio of “low-DA types” (the combination of GG of rs4680 and AA of rs3756450) was significantly lower in the non-TRS group (Table 5). This difference was even more evident in the two-group (TRS and non-TRS groups) comparison: the TRS group had a higher ratio of low-DA types than the non-TRS group.

Catechol-*O*-methyltransferase gene has been shown to be related to multiple symptom domains as well as cognitive impairment in schizophrenia patients (82, 83). Most of the relevant studies have consistently shown that patients with the Val allele (leading to low DA) have severe psychopathology or poor cognitive function (84–87), although a meta-analysis did not support a relationship with working memory (88). In many areas of the human brain such as the striatum, thalamus, and hippocampus, both COMT and DAT provide DA clearance (89, 90), and it has been indicated that DAT could be involved more closely than COMT in the synaptic clefts (91–93). In the prefrontal cortex where DAT is less abundant, DA is broken down by COMT and other transporters such as norepinephrine transporter, and DAT plays a role in the volume transmission of DA away from the synaptic clefts (94, 95), suggesting that the effect of a specific genotype on brain function might differ depending on the brain region.

Several fMRI studies used the N-back task performed by healthy subjects, and with the classification of their genotypes the authors reported that the interacting effect of *COMT* (rs4680) and *SLC6A3* (40-bp VNTR) on brain activation was linear in the dorsolateral prefrontal cortex (96–98), whereas the effect was nonlinear (i.e., an inverted-U-shape curve) in the hippocampus (97). Notably, the lowest-DA genetic combination (defined as the Val allele of *COMT* and the 10-repeat of *SLC6A3*) did not exhibit the lowest DA signal or the highest blood oxygen level-dependent (BOLD) signal, indicating the veracity of the inverted-U-shape. It has been speculated that DA in the prefrontal cortex is modulated in a multi-layered way at both the synaptic level (i.e., by COMT) and the network level in the cortical-striatal-thalamus-cortex pathway (i.e., by both COMT and DAT) (99), leading to differential relationships between molecule functions and brain activation.

Our results after the selection of the 5′-UTR on *SLC6A3* instead of the 40-bp VNTR in the 3′-UTR, demonstrated that the 5′-UTR (rs3475860) alone and the combination of 5′-UTR on *SLC6A3* and *COMT* could be significantly related to TRS (Table 5). Our findings might support a linear interacting effect of *COMT* and *SLC6A3* on the responsiveness to antipsychotic(s) in the striatum, in contrast to the nonlinear interaction (inverted U-shape curve) in the prefrontal cortex under cognitive tasks shown in fMRI studies.

Although several PET studies have reported that DA synthesis is not enhanced in individuals with TRS (24, 25), our present analyses showed that the two SNPs on the *TH* gene were not significantly associated with assignment to the TRS group, or the non-TRS group, or to schizophrenia patients as a whole. There are several potential reasons for this discrepancy between our result and those of the PET studies. Since no SNP with a significant impact on the expression of TH has been found to date, it is possible that a polymorphism other than the two SNPs we selected (rs10770141 and rs6356) may have a greater impact on the enzyme’s function (100, 101). Significant associations of rs10840491, rs10840489, rs11042978, and rs11564717 on *TH* gene in individuals with schizophrenia were reported in association studies of schizophrenia (102, 103), but other reports denied these associations. The synthesis of DA by TH may be influenced by the methylation of *TH* or another upstream region of this molecule (104). In addition, the degree of DA synthesis in patients with TRS observed in the PET studies (i.e., a lack of enhanced DA synthesis) is similar to that in healthy subjects, and in this sense, this finding is not inconsistent with our present results.

Our analyses considering the subjects’ clinically related measures revealed that among the non-TRS patients, the patients with a low-DA type showed only slightly but nevertheless significantly lower values than the patients with other types in terms of psychopathology and antipsychotic doses, in both the single analysis of rs3756450 and the analysis combining rs4680 and rs3756450 (Supplementary Table S3; Table 6). These results in the non-TRS group were in contrast to the TRS-group findings showing that the low-DA type(s) could be risk genotypes for TRS with severe psychopathology (Table 5). However, the results concerning the non-TRS group were not contradictory since patients in the non-TRS group respond to antipsychotic medication by definition: the schizophrenia of the patients with the low-DA types in particular would be controlled with lower doses of antipsychotics, and these patients would thus experience better social lives, including employment.

This study has several strengths over other similar association studies of TRS patients. The sample size was relatively large. In addition, the majority of the prior similar studies reported that the diagnosis of TRS was based on a threshold antipsychotic dosage (e.g., >400 or > 600 mg), whereas we conducted a thorough review of the patients’ medical records to determine the diagnosis of TRS. The results of that review also reflect the patients’ clinical courses to the greatest extent possible, and the validity of the TRS diagnosis is high, which is another study strength.

However, there are several study limitations to address. The first is that although the sample size is relatively large, it was still small enough to have insufficient statistical power. A second limitation is that the patients’ responses to antipsychotic medication were not evaluated with standard assessment tools such as the Positive and Negative Syndrome Scale (PANSS) or the Brief Psychiatric Rating Scale (BPRS). Patients with TRS are heterogeneous in their response to antipsychotic(s) and other non-genetic factors such as adherence to medication and the duration of untreated psychosis (4, 5). Several research groups recently proposed that TRS could be classified into an early subtype and a late subtype (and others), and the late subtype of TRS is speculated to be related to the development of dopamine supersensitivity psychosis (DSP) (5). Patients with DSP respond well to initial antipsychotics for their first episode of psychosis by definition, and thus the dopaminergic function of this subtype could be similar to that of non-TRS patients, but not to that of an early subtype of TRS. However, we could not collect data relevant to DSP for all of the present patients, and we did not performed such an analysis of DSP in the present study. To more precisely examine the relationships between genetic-based phenotypes and responsiveness to medication even among only patients with TRS, more objective and thorough assessments are necessary.

In addition, our analysis strategy might be slightly arbitrary; we did not measure DA in the patients’ brains, and relationships between each variant and their presumed DA level are only hypothetical, particularly in the analysis of the *COMT* and *DAT* combination, which did not reflect the actual DA state in brains. Lastly, the targeted genes and polymorphisms were greatly restricted. Other genes (e.g., *MAO-B* and *VMAT2*) or functional/non-coding variants could have influenced our findings. Resequencing analyses for the relevant genes are required to obtain more comprehensive results.

In conclusion, the results of our analyses revealed significant difference in allelic and genotypic distributions of rs3756450 between schizophrenia patients with and without a treatment-resistant phenotype. These results partially support those of several PET studies showing non-enhancement of the DA synthesis pathway in patients with TRS. Our analysis of the *COMT* rs4680 and *SLC6A3* rs3756450 combination suggests that a presumptive low-DA state is more frequently present in the TRS patients compared to non-TRS patients. These findings suggest that DA modulation in the synaptic cleft by DAT could affect patients’ responses to antipsychotics medication, ultimately leading to the development of TRS.

## Data availability statement

The datasets presented in this study can be found in online repositories. ClinVar (the accession number 509431) can be found here: https://www.ncbi.nlm.nih.gov/clinvar/submitters/509431.

## Ethics statement

The studies involving humans were approved by the Biomedical Research Ethics Committee of the Graduate School of Medicine of Chiba University. The studies were conducted in accordance with the local legislation and institutional requirements. Written informed consent for participation in this study was provided by the participants or thier legal guardians.

## Author contributions

MKo: Conceptualization, Data curation, Formal Analysis, Funding acquisition, Investigation, Methodology, Project administration, Resources, Validation, Visualization, Writing – original draft, Writing – review & editing. NK: Conceptualization, Data curation, Formal Analysis, Funding acquisition, Investigation, Methodology, Project administration, Resources, Supervision, Validation, Visualization, Writing – original draft, Writing – review & editing. AM: Investigation, Resources, Writing – review & editing. YS: Formal Analysis, Methodology, Software, Writing – review & editing. IO: Investigation, Resources, Writing – review & editing. KM: Investigation, Resources, Writing – review & editing. MTak: Investigation, Resources, Writing – review & editing. MKi: Investigation, Resources, Writing – review & editing. HK: Investigation, Resources, Writing – review & editing. KO: Investigation, Resources, Writing – review & editing. KI: Investigation, Resources, Writing – review & editing. MTam: Investigation, Resources, Writing – review & editing. YO: Investigation, Resources, Writing – review & editing. TY: Investigation, Resources, Writing – review & editing. SO: Investigation, Resources, Writing – review & editing. FY: Investigation, Resources, Writing – review & editing. YN: Investigation, Resources, Writing – review & editing. YW: Investigation, Resources, Writing – review & editing. TN: Investigation, Resources, Writing – review & editing. AH: Conceptualization, Investigation, Resources, Writing – review & editing. MI: Conceptualization, Investigation, Resources, Visualization, Writing – review & editing.
