# Supplementary material for: Association of SLC6A3 variants with treatment-resistant schizophrenia: a genetic association study of dopamine-related genes in schizophrenia
Source: Front Psychiatry. 2024 Feb 27;14:1334335. doi: 10.3389/fpsyt.2023.1334335 (PMC10929739; doi:10.3389/fpsyt.2023.1334335)
Supplement: Supplementary file 1 [file Data_Sheet_1.docx]

**Suppl. Table S1. Comparisons of the allelic and genotype distributions of the eight studied SNPs between the schizophrenia group and the healthy control group**

| **SNP** | **Major/minor**  **allele [MAF]** ^#^ | **Group** | **Genotype n [%]** | | | | **Allele n [%]** | | | | **OR** | **95%CI** | |
| --- | --- | --- | --- | --- | --- | --- | --- | --- | --- | --- | --- | --- | --- |
|  |  |  | **MM** | **Mm** | **mm** | **p-value** | **M** | **m** |  | **p-value** |  |  |  |
| ***TH*** | | | | | | | | | | | | | |
| rs10770141 | G > A  [A:0.068] | HC | 359 [85.7] | 57 [13.6] | 3 [0.7] | 0.732 | 775 [92.5] | 63 [7.5] |  | 0.564 | 0.912 | 0.668-1.246 | |
|  |  | SCH | 828 [86.6] | 124 [13.0] | 4 [0.4] |  | 1780 [93.1] | 132 [6.9] |  |  |  |  |  |
| rs6356 | A > G  [G:0.325] | HC | 201 [49.4] | 170 [41.8] | 36 [8.8] | 0.942 | 572 [70.3] | 242 [29.7] |  | 0.734 | 1.032 | 0.862-1.235 | |
|  |  | SCH | 453 [48.6] | 393 [42.1] | 87 [9.3] |  | 1299 [69.6] | 567 [30.4] |  |  |  |  |  |
| ***DRD2*** | | | | | | | | | | | | | |
| rs1800497 | G > A  [A:0.373] | HC | 205 [45.7] | 184 [41.0] | 60 [13.4] | 0.184 | 594 [66.1] | 304 [33.9] |  | 0.152 | 1.129 | | 0.956-1.334 |
|  |  | SCH | 390 [40.6] | 438 [45.6] | 133 [13.8] |  | 1218 [63.4] | 704 [36.6] |  |  |  |  |  |
| rs6275 | A > G  [G:0.432] | HC | 176 [37.1] | 215 [45.3] | 84 [17.7] | 0.262 | 567 [59.7] | 383 [40.3] |  | 0.268 | 1.094 | | 0.934-1.281 |
|  |  | SCH | 315 [32.9] | 472 [49.3] | 171 [17.8] |  | 1102 [57.5] | 814 [42.5] |  |  |  |  |  |
| ***C COMT*** | | | | | | | | | | | | | |
| rs4680 | G > A  [A:0.317] | HC | 205 [42.8] | 210 [43.8] | 64 [13.4] | 0.209 | 620 [64.7] | 338 [35.3] |  | 0.144 | 0.885 | | 0.752-1.042 |
|  |  | SCH | 435 [45.2] | 429 [44.5] | 99 [10.3] |  | 1299 [67.4] | 627 [32.6] |  |  |  |  |  |

**Suppl. Table S1 (*Continued*)**

| **SNP** | **Major/minor**  **allele [MAF]** ^a^ | **Group** | **Genotype n [%]** | | | | **Allele n [%]** | | | | **OR** | **95%CI** |
| --- | --- | --- | --- | --- | --- | --- | --- | --- | --- | --- | --- | --- |
|  |  |  | **MM** | **Mm** | **mm** | **P-value** | **M** | **m** |  | **p-value** |  |  |
| ***SLC6A3*** | | | | | | | | | | | | |
| rs3756450 | A > G  [G:0.469] | HC | 133 [30.8] | 210 [48.6] | 89 [20.6] | 0.180 | 476 [55.1] | 388 [44.9] |  | 0.070 | 1.163 | 0.988-1.369 |
|  |  | SCH | 236 [26.3] | 450 [50.1] | 212 [23.6] |  | 922 [51.3] | 874 [48.7] |  |  |  |  |
| rs420422 | G > A  [A:0.392] | HC | 152 [35.8] | 202 [47.6] | 70 [16.5] | 0.201 | 506 [59.7] | 342 [40.3] |  | 0.076 | 0.860 | 0.728-1.016 |
|  |  | SCH | 371 [39.9] | 433 [46.6] | 125 [13.5] |  | 1175 [63.2] | 683 [36.8] |  |  |  |  |
|  |  |  | **10 10** | **10 X** | **X Y** |  | **10 repeat** | **9 repeat** | **Others** |  |  |  |
| 40-bp VNTR | 10 > others  [10: 0.93] | HC | 93 [91.2] | 9 [8.8] | 0 [0] | **0.021** | 195 [95.6] | 5 [2.5] | 4 [2.0] | **0.014** | 2.864 | 1.373-5.976 |
|  |  | SCH | 156 [79.2] | 36 [18.3] | 5 [2.5] |  | 348 [88.3] | 23 [5.8] | 23 [5.8] |  |  |  |

^a^The MAF data were obtained from the U.S. National Library of Medicine. VNTR: variable number of tandem repeats.

#. from Sano et al. [1993]

**Suppl. Table S2. Comparisons of the genotype distributions of the 40-bp VNTR between the schizophrenia group and the healthy control group**

|  | **Group** | **Repeat n [%]** | | | |
| --- | --- | --- | --- | --- | --- |
|  |  | 10 repeats | 9 repeats | others | **p-value** |
| 40bp-VNTR | HC | 195 [95.6] | 5 [2.5] | 4 [2.0] | **0.023** |
|  | non-TRS | 177 [89.8] | 8 [4.1] | 12 [6.1] |  |
|  | TRS | 171 [86.8] | 15 [7.6] | 11 [5.6] |  |

**Suppl. Table S3.** **Comparison of clinically related measures for low-DA and other genotypes in the non-TRS groups**

| **Variables** | **rs4680/rs3756450: GG/AA n=42** | **Others**  **n=458** | **Statistic values** |
| --- | --- | --- | --- |
| **﻿Sex: male/female [n]** | 25 / 17 | 218 / 239 | χ2=0.010, p=0.921 |
| **Age at time of blood sampling [yrs]** | 26.00 (9.45) | 27.31 (10.11) | *t*=0.613, p=0.540 |
| **Age at onset [yrs]** | 12.78 (9.32) | 14.33 (12.45) | *t*=0.582, p=0.561 |
| **Duration of disease [yrs]** | 45.26 (15.70) | 45.04 (15.12) | *t*=0.089, p=0.930 |
| **CGI-S** | 2.58 (0.88) | 3.17 (1.17) | *t*=2.399, **p=0.017** |
| **Number of hospitalizations [times]** | 1.48 (1.90) | 1.40 (1.96) | *t*=0.203, p=0.840 |
| **Work experience following disease onset: Yes/No** | 22 / 2 | 150 / 123 | χ2=12.205, **p<0.001** |
| **Employment at time of blood sampling: Yes/No** | 16 / 7 | 89 / 186 | χ2=12.872, **p<0.001** |
| **ECT from onset of illness to time of blood sampling Yes/No** | 2 / 23 | 7 / 282 | χ2=2.653, p=0.103 |
| **Clozapine medication from onset to present: Yes/No** | 0 / 25 | 0 / 287 | - |
| **Antipsychotic dose (CP-eq.) [mg]** | 313.1 (173.4) | 453.3 (320.1) | *t*=2.203, **p=0.028** |
| **Monotherapy with antipsychotic: Yes/No** | 23 / 3 | 230 / 58 | χ2=1.127, p=0.288 |

Sex was unknown for 1 patient in the others group.
